# Crosstalk Between Programmed Death Ligand 1, Ki-67 Labelling Index, and Tumor-Infiltrating Lymphocytes in Invasive Breast Cancer and Clinicopathological Correlations in a Tertiary Care Center in Western India

**DOI:** 10.30699/IJP.2022.539946.2737

**Published:** 2022-08-13

**Authors:** Rashim Sharma, Poonam Abhay Elhence, Meenakshi Rao, Sudeep Khera, Deepak Vedant, Ramkaran Chaudhary, Puneet Pareek, Jeewan Ram Vishnoi, Sanjeev Misra

**Affiliations:** 1 *Department of Pathology and Laboratory Medicine, All India Institute of Medical Sciences, Jodhpur, Rajasthan, India*; 2 *Department of General Surgery, All India Institute of Medical Sciences, Jodhpur, Rajasthan, India*; 3 *Department of Radiation Oncology, All India Institute of Medical Sciences, Jodhpur, Rajasthan, India*; 4 *Department of Surgical Oncology, All India Institute of Medical Sciences, Jodhpur, Rajasthan, India*

**Keywords:** Breast Carcinoma, Chemotherapy, Estrogen Receptors, Ki-67, Programmed Death Ligand 1, Tumor-infiltrating Lymphocytes

## Abstract

**Background & Objective:**

Breast cancer is the leading cancer among Indian women and accounts for about 25% of all cancer cases worldwide. The present study aimed to assess Programmed Death Ligand-1 (PD-L1) expression in tumoral cells and tumor-infiltrating lymphocytes (TILs) and evaluate their correlations with the Ki-67 labelling index in invasive breast carcinomas (IBC).

**Methods:**

This descriptive observational study was conducted during 2016-2018 and included all diagnosed cases of IBC. The relationships between PD-L1 expression, TILs, hormone receptors, Ki-67, and clinicopathological parameters were studied in IBC. Statistical analysis was performed by SPSS version 23.

**Results:**

Out of 114 evaluated cases, 33.33% (N=38) showed PD-L1+ expression in tumor cells and 47.37% (N=54) had PD-L1+ expression in TILs. A high Ki-67 index was observed in 96 cases. Moreover, 49 patients were estrogen receptor (ER)- and 65 were ER+. We observed that 22 of 49 ER- and 49 of 65 ER+ subjects showed PD-L1+ expression, respectively.

**Conclusion:**

Our results showed a significant relationship between PD-L1 expression in tumoral cells and TILs, as well as between Ki-67 and TILs. In addition, an inverse correlation was noted between PD-L1 expression and ER. The PD-L1 expression in tumors and TILs and correlation with high Ki-67 may prove the importance of PD-L1 in targeted chemotherapy. An inverse relationship between PD-L1 and ER expression in tumoral cells suggests scope for immunotherapy in ER- IBC. However, further research with more cases is required.

## Introduction

Breast cancer is the most common cancer affecting women and accounts for approximately one-fourth of all cancers (1). There is an increasing trend in the incidence of breast cancer, cancer morbidity, and mortality in India. It ranks as the first cancer among all Indian females, with an age-adjusted rate being 25.8 per 100,000 women and a mortality rate of 12.7 per 100,000 women (1, 2). Performing core biopsies of palpable breast lumps and enlarged axillary lymph nodes has significantly bypassed the requirement of open surgical biopsy. In addition, assessing the expression of estrogen receptor (ER), progesterone receptor (PgR), and Her-2/neu has facilitated targeted therapy in invasive breast carcinoma (IBC). Programmed Death Ligand 1 (PD-L1) is a 40 kDa transmembrane protein found on epithelial cells, macrophages, vascular endothelial cells, dendritic cells, natural killer cells, and B-cells (3). The mechanism of its action has been studied in various immune diseases and cancers. The PD-1 by binding with its ligand PD-L1 downregulates the activation of T-cells (4). This binding also enhances tumorigenesis by blocking T-cell expression. The PD-L1 serves as a backdoor escape mechanism for tumoral cells and, in turn, is overexpressed in cancer. The PD-L1 expression can be easily studied by immunohistochemistry (IHC) as membranous staining. The present study aimed to ascertain a relationship between the expression of PD-L1 in the tumor, TILs, ER, PgR, and Her-2/neu. Furthermore, the Ki-67 labelling index in IBC was compared with expression in TILs. This study also evaluated the relationship between PD-L1 expression and clinical parameters.

## Material and Methods

**Study Population**

**Fig. 1:**
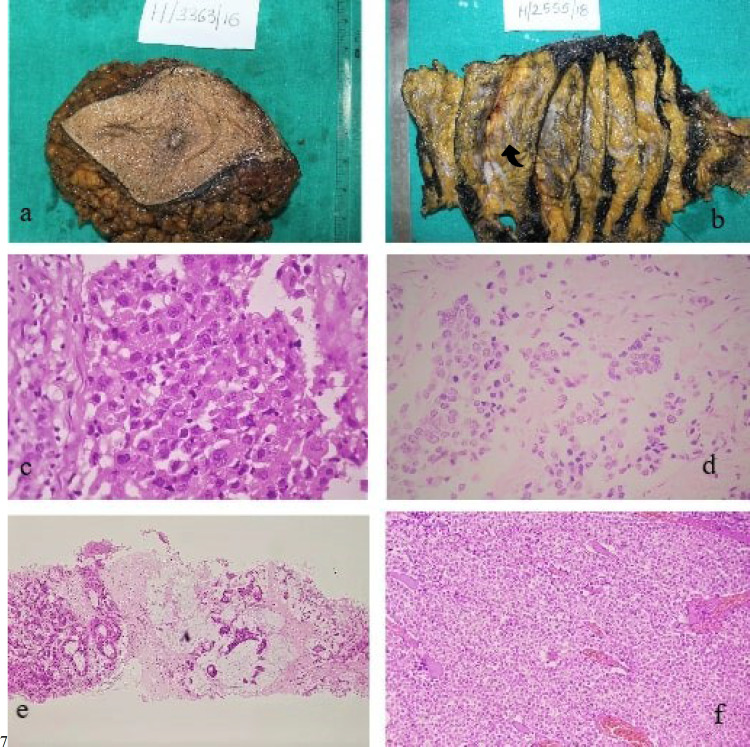
(a-f). a: Gross photograph of breast carcinoma; b: Left MRM, serially sliced showing tumor in lower inner quadrant marked with arrowhead; c: High-grade invasive breast carcinoma, H&E, ×40; d: Low-grade invasive breast carcinoma, H&E, ×40; e: Mucinous carcinoma breast, H&E, ×10; f: Invasive lobular carcinoma breast, H&E, ×10

All patients diagnosed with IBC were included in the current study. A total of 114 IBC samples were received during January 2016-December 2018 in the Department of Pathology and Laboratory Medicine, All India Institute of Medical Sciences (AIIMS), Jodhpur, Rajasthan, India after fulfilling the inclusion and exclusion criteria. Cases with benign or premalignant breast lesions, cases with a prior history of radiotherapy/chemotherapy for other malignancies, and inadequate samples were excluded from the study. Core biopsies, lumpectomies, quadrantectomies, and modified radical mastectomy (MRM) were received and all the relevant clinical details were collected. Few representative gross images of MRM with photomicrographs of low-grade and high-grade IBC, mucinous carcinoma, and lobular breast carcinoma are shown in Figure 1. 

**Tumor Samples and Immunohistochemistry**

One representative tumor section was selected for IHC from each case. The IHC for PD-L1 (Clone: CAL 10, ready to use, Rabbit Monoclonal Antibody, Biocare Medical, California, USA), Ki-67 (Clone: SP-6, Rabbit Monoclonal Antibody, Thermo Fisher Scientific, Waltham, Massachusetts, USA), ER (Clone: SP-1, Rabbit Monoclonal Antibody, Thermo Fisher Scientific, Waltham, Massachusetts, USA), PgR (Clone: SP-2, Rabbit Monoclonal Antibody, Thermo Fisher Scientific, Waltham, Massachusetts, USA), and Her-2/neu (Clone: EP-3, Rabbit Monoclonal Antibody, PathnSitu, Pleasanton, California, USA) was applied along with the relevant positive external controls. 

Semiquantitative analysis of PD-L1, ER, and PgR was completed, and the Allred score was given as per intensity and proportion staining (5, 6). The membranous expression of PD-L1 was noted. The Ki-67 nuclear positivity was noted, and percentage was calculated in the tumoral cells according to the recommendations of the International Ki-67 Breast Cancer Working Group (7). Her-2/neu was assessed based on ASCO/CAP guidelines, 2018 (8). The TILs were assessed in Hematoxylin and Eosin (H&E) stained slides according to the recommendations of the International TILs Working Group, 2014 (9). The PD-L1 expression in the tumoral cells and TILs and total effect on both was studied and correlated with factors, such as age, hormone receptor status, and lymph node metastases. A similar section of the tissue was chosen for scoring the percentage of TILs and applying IHC. 

**Statistical Analysis**

The data were entered in Excel and were analyzed by SPSS version 23 (IBM Co., Armonk, NY, USA). Chi-squared and Fisher’s exact tests were used to analyze the expression of PD-L1 in tumors and TILs and evaluate its possible association with the existing biomarkers ER, PgR, and Her-2/neu. The TILs were quantified and compared with the Ki-67 labelling index.

## Results

**Characteristics of Patients and Tumors**

Out of 114 cases, 112 were women and 2 were men. We observed that 28 patients had received pre-op neoadjuvant chemotherapy (NACT), whereas 86 cases did not receive any NACT (summarized in Table 1). Most of the patients with IBC were in the age group of 51-60 years (28.07%) (Tables 1 and 2). 

**Table 1:** Clinical parameters, hormonal biomarkers, tumor grade, Ki-67 labelling index, lymph node metastases, extra nodal extension, and pTNM status

Group	Feature		Frequency	Percentage
Age (years), N=114	20-60		81	**71.05**
	Elderly (>60)		33	**29.84**
Gender, N=114	Female		112	**98.25**
	Male		02	**1.75**
Menstrual history (N=112)	Pre-menopausal		45	**40.18**
	Post-menopausal		67	**59.82**
Clinical features (N=114)	Pain		39	**34.21**
	Pain, lump		75	**65.79**
Chemotherapeutic intervention (N=114)	Yes		28	**24.56**
	No		86	**75.44**
Hormonal biomarkers (N=114)	ER	Positive	65	**57.02**
		Negative	49	**42.98**
	PgR	Positive	49	**42.98**
		Negative	65	**57.02**
	Her2/ Neu	Positive	47	**41.23**
		Negative	56	**49.12**
		Equivocal	11	**9.65**
Histological tumor grade (N=86)	Grade 1		11	**9.65**
	Grade 2		58	**50.88**
	Grade 3		17	**14.91**
Ki-67 labelling index (N=114)	<14		18	**15.79**
	≥14		96	**84.21**
Lymph node metastases (N=63)	Positive		42	**67.74**
	Negative		21	**33.33**
Extranodal extension (ENE) (N=63)	Positive		13	**20.63**
	Negative		50	**79.37**
pTNM status (N=35)	pT1cN0		1	**2.86**
	pT2N0		15	**42.86**
	pT2N1a		6	**17.14**
	pT2N2a		4	**11.43**
	pT3N1a		2	**5.71**
	pT3N2a		2	**5.71**
	pT2N3a		2	**5.71**
**pT3N3a**		**3**	**8.57**

**Table 2:** Age distribution

Age group	Frequency	Percentage
20-30	03	**2.63**
31-40	16	**14.04**
41-50	30	**26.32**
51-60	32	**28.07**
61-70	26	**22.8**
71-80	06	**5.26**
81-90	0	**0**
91-100	01	**0.88**
Total	**114**	**100**

**PD-L1 Expression in Tumoral Cells and TILs**

A statistically significant relationship was noted between PD-L1 expression in the tumoral cells and TILs (Table 3). Our results showed that 33.33% of patients (38 cases) had PD-L1 expression in the tumoral cells and 47.37% (54 cases) had PD-L1 expression in TILs. The PD-L1 helps in tumor escape, implying that without chemotherapeutic intervention, the tumor has the potential to bypass the immune system, progress, and become poorly differentiated. There was a significant association between TILs on H&E and the Ki-67 labelling index (N=114) (Table 4). Therefore, it was suggested that the more proliferative activity a tumor exhibits, the higher the number of TILs to restrain it. In addition, the present study indicated 47.37% cases with PD-L1 expression in TILs implying a purported greater benefit by targeted chemotherapy and in suppressing the tumor cell population.

**Table 3:** Correlation of PD-L1 expression in tumor and TILs (N=114)

			PD-L1 TILs		Total
			Negative	Positive	
PD-L1 tumor	Negative	Count	53	23	**76**
		% within PD-L1 TILs	88.3%	42.6%	**66.7%**
	Positive	Count	7	31	**38**
		% within PD-L1 TILs	11.7%	57.4%	**33.3%**
Total		Count	60	54	**114**
**% within PD-L1 TILs**	**100%**	**100%**	**100%**

*P*<0.001 by Chi-squared test

**Table 4:** Correlation of Ki-67 labelling index and sTILs (H&E) (N=114)

			Ki67 (%)		Total
			<14%	>14%	
H&E sTILs categorization	Low (0-10%)	Count	15	43	**58**
		Ki67 (%)	83.3%	44.8%	**50.9%**
	Intermediate or High (>10%)	Count	3	53	**56**
		Ki67 (%)	16.7%	55.2%	**49.1%**
Total		Count	18	96	**114**
**Ki67 (%)**	**100%**	**100%**	**100%**

*P*=0.003 by Chi-squared test

**IHC for ER, PgR, Her-2/neu, and PD-L1**

**Fig. 2:**
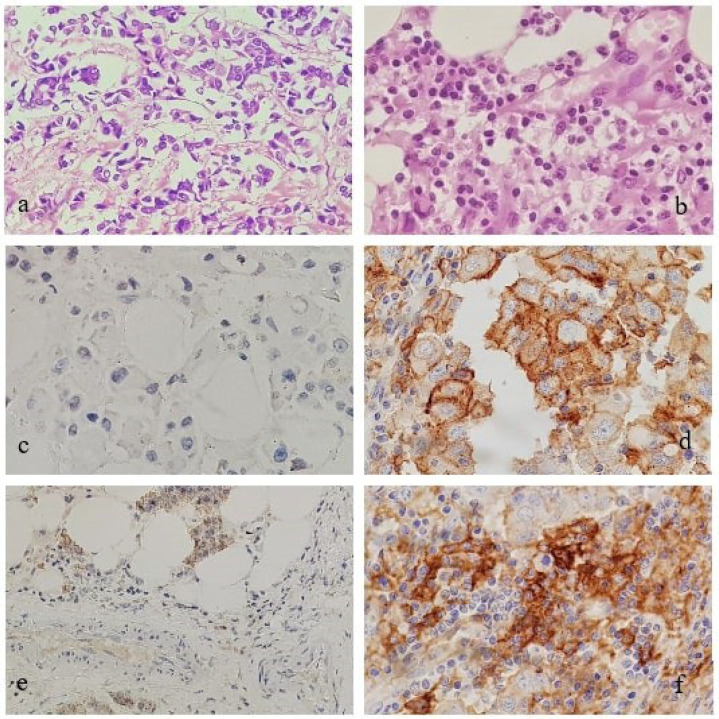
(a-f). a: Low TILs, H&E, ×40; b: High TILs, H&E, ×40; c: Low/absent PD-L1 expression in invasive breast carcinoma, ×40; d: Membranous PD-L1 expression in invasive breast carcinoma, ×40; e: Low/absent PD-L1 expression in TILs in invasive breast carcinoma, ×20; f: Membranous PD-L1 expression in TILs in a case of invasive breast carcinoma, ×40

Results of IHC for ER and PgR were assessed as positive/negative based on the Allred scoring. Quantification of TILs (H&E), membranous staining of PD-L1 IHC in tumors, and TILs are shown in Figure 2. The ER and PD-L1 expression had a significant inverse relationship (Table 5). This finding suggested the possibility of using immunotherapy for PD-L1 in ER- cases. No statistically significant association was noted between PD-L1 expression, PgR, Her-2/neu, and clinicopathological parameters, namely age, clinical features, menstrual history, tumor grade, tumor size, pTNM, lymph node metastases, and extranodal extension. However, PD-L1 expression had a statistically significant correlation with triple-negative breast cancer (TNBC), but the results could not be validated due to the small sample size (N=24).

**Table 5:** Correlation of ER with PD-L1 expression in the tumors (N=114)

Correlation of ER with PD-L1 expression in tumor (N=114)					
			PD-L1 tumor		Total
			Negative	Positive	
ER	Negative	Count	27	22	**49**
		% within PD-L1 tumor	35.5%	57.9%	**43.0%**
	Positive	Count	49	16	**65**
		% within PD-L1 tumor	64.5%	42.1%	**57%**
Total		Count	76	38	**114**
**% within PD-L1 tumor**	**100%**	**100%**	**100%**

## Discussion

Breast cancer detection and treatment have evolved a lot in the last two decades. Cancer therapy has undergone a revolution due to the development of immune checkpoint inhibitors. The CTLA-4 and PD-1 are two negative regulatory proteins that share a 33% amino acid match. They suppress the co-stimulatory molecule by either binding to it or its own ligand and downregulate and eventually suppress the immune system (10-12). The PD-1 is a B7 family negative regulatory molecule on T, B, and myeloid cells which facilitates immune tolerance by causing cellular anergy following binding one of its ligands PD-L1 (11). The PD-L1 and PD-L2 are also expressed in other non-lymphoid organs (11, 13). Consequently, targeted therapies against CTLA-4 or PD-1/PD-L1 signaling pathways can rejuvenate the anti-tumor response and generate a good clinical response (14). The present study described the role of PD-L1 in carcinogenesis. Our findings (Tables 2, 3, and 4) were in line with the results of Schalper* et al.* who suggested an association between higher PD-L1 expression and raised TILs, leading to better recurrence-free survival in breast cancer patients (15). Velcheti* et al.* studied the expression of PD-L1 in non-small cell lung carcinoma cases and found similar results (16). Moreover, consistent findings were observed in breast carcinoma by Bae* et al.* and Wimberly* et al.* (4, 17). Miyoshi* et al.* also suggested a robust association between high TILs and higher Ki-67 labelling index, which corroborated and reinforced the findings of the present study. These results proposed the utility of TILs as a predictive marker because accretion of TILs in the vicinity of tumor cells shows vulnerability yet the aggressiveness of the tumor cell population, necessitating a novel targeted chemotherapy (18). Evangelou* et al.* conducted research on women under 40 years of age and confirmed similar findings on PD-L1 and Ki-67 labelling index (19).

Liu* et al.* performed studies on cell lines and segregated ER+ and ER- cases and reported an inversely proportional relationship between ER- tumor cells and PD-L1+ expression. Similar results were found in the current study (Table 5), except that we did not use cell lines (20). Ghebeh* et al.* proposed an inverse association between these two parameters. As ER+ breast carcinoma responds better to chemotherapy, they postulated that ER- status and PD-L1+ expression in the tumor may lead to tumor evasion and may be considered a poor prognostic indicator for patients (21). The present investigation indicated that the expression of PD-L1 in the tumoral cells did not have a significant correlation with PgR and Her-2/neu. This was in contrast to the results of Ghebeh* et al.,* who demonstrated that PD-L1 expression in the tumor was significantly correlated with PgR- and Her-2/neu+ breast cancers (21). Sabatier* et al.* also had similar findings to Ghebeh* et al.* (22). Yuan* et al.* observed that PD-L1 expression in tumors did not have a significant correlation with ER/PgR and Her-2/neu expression (23). 

The present study showed no statistically significant association of PD-L1 expression with clinicopathological parameters. This was in agreement with studies by Kim* et al.*, Lou* et al.,* and Li* et al.* (24-26). Kim* et al.* revealed that PD-L1 expression in the tumor was not significantly associated with age, tumor size, histological grade, and lymph node metastases (24). Lou* et al.* and Li* et al.* showed that PD-L1 expression in the tumors had no significant association with age, menstrual history, tumor diameter, and lymph node metastases (25, 26). 

However, Yuan* et al.* noted a discordant expression of PD-L1 in primary breast cancer and paired axillary lymph nodes (23). Ming Li* et al.* noted that the expression of PD-L1 in the lymph node metastases was higher than in paired breast cancer and hence, they suggested that the expression of PD-L1 protein was better analyzed in the lymph nodes (27). Alves* et al.* investigated PD-L1 expression in primary breast cancer and the lymph nodes and suggested PD-L1 expression in the lymph node metastases, but unrelated to primary breast cancer clinicopathological features (28). Therefore, large-scale studies of PD-L1 expression in primary cancer as well as paired lymph nodes were suggested. Targeted chemotherapy with PD-1 and PD-L1 inhibitors, pembrolizumab, and atezolizumab has widened the horizon in breast cancer treatment (29). Qi* et al.* explored the efficacy and safety of PD-1 and PD-L1 in metastatic breast cancer and found PD-1/PD-L1 monotherapy reliable and suggested better clinical efficacy in cases with high PD-L1 expression (30). 

## Conclusion

The PD-L1 expression in IBC could be a suitable marker for immunotherapy. The PD-L1 expression has a direct relationship with the Ki-67 labelling index and an inverse relationship with ER expression in the tumoral cells. As a result, we suggest that IBC cases that are ER- can benefit from immunotherapy. The TNBC cases have a worse prognosis, while PD-L1 targeted therapy may prove beneficial even in such cases. Furthermore, TILs had a statistically significant association with PD-L1 in the tumoral cells, suggesting the immune capacity of the host against the rapidly increasing the tumor burden. Large-scale studies are required to further establish PD-L1 marker expression in IBC and its benefit for targeted treatment in the cohort of IBC cases which are ER-.

## Declarations

Funding: Not applicable. 

## Conflicts of Interest/Competing Interests

The authors declare no conflict of interest.

Availability of data and material: all data and material of this research have been incorporated into the manuscript.

## Authors' Contribution:

Conceptualization: Rashim Sharma, Poonam Abhay Elhence; Methodology: Rashim Sharma, Poonam Abhay Elhence, Meenakshi Rao, Sudeep Khera, Deepak Vedant, Ramkaran Chaudhary, Jeewan Ram Vishnoi, Puneet Pareek; Formal analysis and investigation: Rashim Sharma, Poonam Abhay Elhence, Meenakshi Rao, Sudeep Khera, Deepak Vedant; Original draft preparation: Rashim Sharma; Review and editing: Rashim Sharma, Poonam Abhay Elhence, Meenakshi Rao, Sudeep Khera, Deepak Vedant; Resources: Rashim Sharma, Poonam Abhay Elhence, Meenakshi Rao, Sudeep Khera, Deepak Vedant, Ramkaran Chaudhary, Jeewan Ram Vishnoi, Puneet Pareek, Sanjeev Misra; Supervision: Poonam Abhay Elhence, Meenakshi Rao, Deepak Vedant.

## Ethics Approval

 All procedures involving human participants were following the ethical standards of the Institutional Ethics Committee, 1964 Helsinki Declaration, and its later amendments or comparable ethical standards. Ethical clearance was granted by Institutional Ethics Committee. Certificate reference number: AIIMS/IEC/2018/451.

## Consent to Participate

 Informed consent was obtained from all participants included in the study.

## Consent for Publication

 Informed consent was obtained from all participants included in the study.
